# Silk Vista Baby Is a Safe and Technically Feasible Flow Diverting Stent for Distal Aneurysm Treatment

**DOI:** 10.3389/fneur.2021.676749

**Published:** 2021-05-12

**Authors:** Vladimir Gavrilovic, Annarita Dapoto, Nicola Marotti, Andrea Pellegrin, Alessandro Pauro, Alessandro Vit, Massimo Sponza

**Affiliations:** Angiography and Interventional Radiology Unit, Department of Radiology, University Hospital of Udine, Udine, Italy

**Keywords:** angiography, endovascular procedures, intracranial aneurysm, flow diverter, magnetic resonance imaging, subarachnoid hemorrhage, thrombosis

## Abstract

**Background and Purpose:** Flow diverting stents are designed to divert blood flow from the aneurysm sac, allowing for eventual occlusion following endovascular therapy. This case series reports clinical experience using the Silk Vista Baby (SVB, Balt Extrusion, Montmorency, France), a flow diverter (FD) designed to treat intracranial aneurysms in small, distal vessels.

**Methods:** All patients who underwent treatment with SVB at the University Hospital “Santa Maria della Misericordia” of Udine between July 2018 and September 2020 were retrospectively identified. Baseline patient and aneurysm characteristics, intraprocedural technical outcomes, periprocedural complications, modified Rankin Scale (mRS) at discharge, magnetic resonance imaging (MRI) results at 3-month follow-up, and angiographic results at 6-month follow-up were collected.

**Results:** A total of 18 patients (55.6% [10/18] male; mean age 62.6 years, range: 42–77 years) were retrospectively identified, receiving treatment for 22 aneurysms. Most patients were symptomatic (14/18, 77.8%) and approximately half had subarachnoid hemorrhage (10/18, 55.6%). Sufficient aneurysm coverage was achieved in 88.9% (16/18) of patients with a single device. Mortality did not occur (0/18, 0%); adverse device-related events included side branch occlusion (1/18, 5.6%) and in-stent thrombosis (1/18, 5.6%). At discharge, 77.8% (14/18) had an mRS of 0. In most cases, patients showed complete occlusion (10/15, 66.7%) or a small aneurysmal remnant (3/15, 20.0%) upon MRI; upon angiography, most showed complete occlusion (10/13, 76.9%) or only a small aneurysmal remnant (2/13, 15.4%).

**Conclusion:** This case series showed that the SVB FD is safe and feasible to use in patients with aneurysms in small, distal vessels. Additional randomized, prospective studies with larger cohorts are needed for the SVB.

## Introduction

Flow diversion is a relatively recent development in endovascular treatment wherein a flow diverter (FD) stent is placed over an aneurysm to disrupt blood flow into the aneurysm sac, thus providing a scaffold for neo-endothelialization and eventual occlusion to occur (1, 2). Flow diversion strategies have gained popularity for cases where coil embolization might be more difficult or likely to fail, such as with large neck side-wall aneurysms, bifurcation aneurysms, or recurrent aneurysms that have already been treated with coil embolization (3–5). To-date, relatively limited data are available on the feasibility, safety, and effectiveness of flow diversion in aneurysms arising from distal, small-caliber arteries; deploying FDs in such vessels comes with the risk that the FD may become elongated, thus increasing its porosity and lowering the chances of intra-aneurysmal thrombosis (6, 7).

The Silk Vista Baby (SVB, Balt Extrusion, Montmorency, France), a newer iteration of the Silk Flow Diverter (SILK, Balt), is designed to treat aneurysms in vessels 1.5–3.5 mm in diameter (8, 9). The SVB consists of 48 braided nitinol tubes filled with platinum and is deliverable through a 0.017-inch catheter (9, 10). Though the SVB has not received approval from the United States Food and Drug Administration (FDA) for aneurysm treatment, it received its first Conformitè Europëenne (CE) mark in 2018 (8), indicating its safety and approval for use in the European Union. This study reports the technical and clinical outcomes of using the SVB in a series of patients with aneurysms, evaluating the safety of the device in terms of intraprocedural and periprocedural complication rates following flow diversion treatment.

## Materials and Methods

### Patient Population and Analysis

This was a single-center, single-arm, retrospective, observational study of consecutive patients treated with the SVB at University Hospital “Santa Maria della Misericordia” of Udine between July 2018 and September 2020. Patient selection for endovascular treatment was performed by a multidisciplinary team of interventional neuroradiologists, neurologists, and neurosurgeons. The treatment decision was based on the clinical status of the patient as well as aneurysm location, morphology, and size. Patients were informed regarding the risks and complications associated with the endovascular treatment; interventions were only performed after obtaining informed consent. For each patient, information was collected regarding demographic data, clinical presentation, aneurysm location and characteristics, intraprocedural outcomes, immediate angiographic and clinical results, and radiological follow-up results at 3- and 6-months post-procedure. All characteristics and outcomes are presented as frequencies and analyzed based on counts, rates, means, and ranges. Patients were considered “elective” if they were treated without using emergency procedures and prepared with standard dual antiplatelet therapy.

### Antiplatelet Therapy

Patients received mandatory dual antiplatelet therapy prior to implantation of FDs on a regimen of 100 mg aspirin and 75 mg clopidogrel twice daily for 5 days. In just one case, the patient with ruptured aneurysm, initially coiled and the next day treated with FD, was loaded with 300 mg of clopidogrel 12 h before the FD treatment and 500 mg of intravenous acetylsalicylated lysine 30 min before stent deployment. For ruptured aneurysms, depending upon the clinical state of the patient, pre-medication was also given immediately before the procedure. Patients were instructed to continue dual antiplatelet therapy for 6 months after the procedure; aspirin is typically continued indefinitely while clopidogrel may be stopped 3 months after the procedure.

### Endovascular Treatment and Follow-Up

All endovascular treatment was performed with the patient under general anesthesia, via right common femoral artery approach, using a bi-axial access system. Interventionalists used a 7F Avanti femoral artery sheath (Cordis, Santa Clara, CA, USA), 6F Envoy guiding catheter (Codman Neuro, Raynham, MA, USA), or 5F Sofia catheter (MicroVention, Aliso Viejo, CA, USA). The Headway 17 soft microcatheter (MicroVention) was used for access to the parent vessel and placement of the SVB FD. The length and diameter of the stent were chosen based on pre- and intra-procedural imaging, which included magnetic resonance imaging (MRI) for evaluation of the aneurysm sac diameter and angiography for evaluation of the vessel diameter. The length of the stent was to be such that the stent extended at least 5 mm beyond both sides of the aneurysm neck, while the diameter of the stent was roughly the diameter of the parent vessel. In this case, interventionalists used SVB that had diameters of 2.25 or 2.75 mm and length of 15 or 20 mm.

All procedures were performed under heparin anticoagulation, with a 5000 IU bolus dose at the start of the procedure and subsequent 1000 IU bolus doses every hour to maintain the activated clotting time at a level of 2–2.5 times the baseline. In addition, administration of nimodipine during the procedure was used to reduce the possibility of vasospasm-related complications (180 ml/h of 2 mg of Nimodipine diluted in 1,000 ml of NaCl 9%). At the end of the endovascular procedure, patients with subarachnoid hemorrhage (SAH) were awakened after 24 h to assess for neurological deficiency, and elective patients were assessed at 72 h post-procedure. At discharge, patients were assessed for neurologic disability using the modified Rankin Scale (mRS) (11). Patients underwent MRI at 3-month follow-up and an angiography exam 6 months after the procedure. Aneurysm occlusion was evaluated using the O'Kelly-Marotta scale (12).

## Results

### Patient Population

During the study period, a total of 18 patients (55.6% [10/18] male; mean age: 62.6 years, range: 42–77 years) were treated for 22 aneurysms. Most patients were symptomatic (14/18, 77.8%); they presented variously with unspecified headache (3/18, 16.7%), dysarthria, hemiplegia, or clonic seizure (9/18, 50.0%), and loss of consciousness (2/18, 11.1%). (It should be noted that the patients who experienced loss of consciousness did so prior to arrival for treatment with SVB. Only patients who could consciously provide informed consent were treated with SVB). Approximately half of the patients (10/18, 5.6%) had SAH and were treated within 90 days of SAH diagnosis. Patient characteristics at baseline are shown in Table 1.

**Table 1:** Patient demographics and baseline characteristics.

	**Frequency**
**Sex**	
Male	10/18, 55.6%
Age (years)	62.6, 42–77
**Symptoms**	
Headache	3/18, 16.7%
Dysarthria, hemiplegia, or clonic seizure	9/18, 50.0%
Unconsciousness	2/18, 11.1%
Subarachnoid hemorrhage	10/18, 55.6%
**Aneurysm characteristics**	
Unruptured	7/22, 31.8%
Acutely ruptured	10/22, 45.5%
Recurrent/previously ruptured	5/22, 22.7%
Saccular	22/22, 100%
Diameter (mm)	8.9, 5–25
**Aneurysm location**	
Anterior circulation	21/22, 95.5%
ACA	4/22, 18.2%
MCA	4/22, 18.2%
PCA	1/22, 4.5%
ACoA	13/22, 59.1%

*Frequencies are reported as (n/N, %) or (mean, range). ACA, anterior cerebral artery; ACoA, anterior communicating artery; MCA, middle cerebral artery; PCA, posterior cerebral artery*.

### Aneurysm Characteristics

Of the 22 aneurysms treated, 31.8% (7/22) were unruptured and 45.5% (10/22) were acutely ruptured. Additionally, 22.7% (5/22) were recurrent and previously ruptured; three of these recurrent aneurysms had been previously treated with coil embolization, and the other two with clip ligation. All aneurysms were saccular, and nearly all (21/22, 95.5%) arose from the anterior circulation. All aneurysms were located on small arteries; specifically, 4 aneurysms (18.2%) involved the anterior cerebral artery (ACA), another 4 aneurysms (18.2%) were located on the middle cerebral artery (MCA), 1 aneurysm (4.5%) was on the posterior cerebral artery (PCA), and 13 aneurysms (59.1%) involved the anterior communicating artery (ACoA). The mean aneurysm diameter was 8.9 mm, with diameters ranging from 5–25 mm. Patients with very small (5–7 mm) aneurysms were treated because they had SAH. A full list of aneurysm characteristics is in Table 1.

### Procedural Characteristics

**Figure 1:**
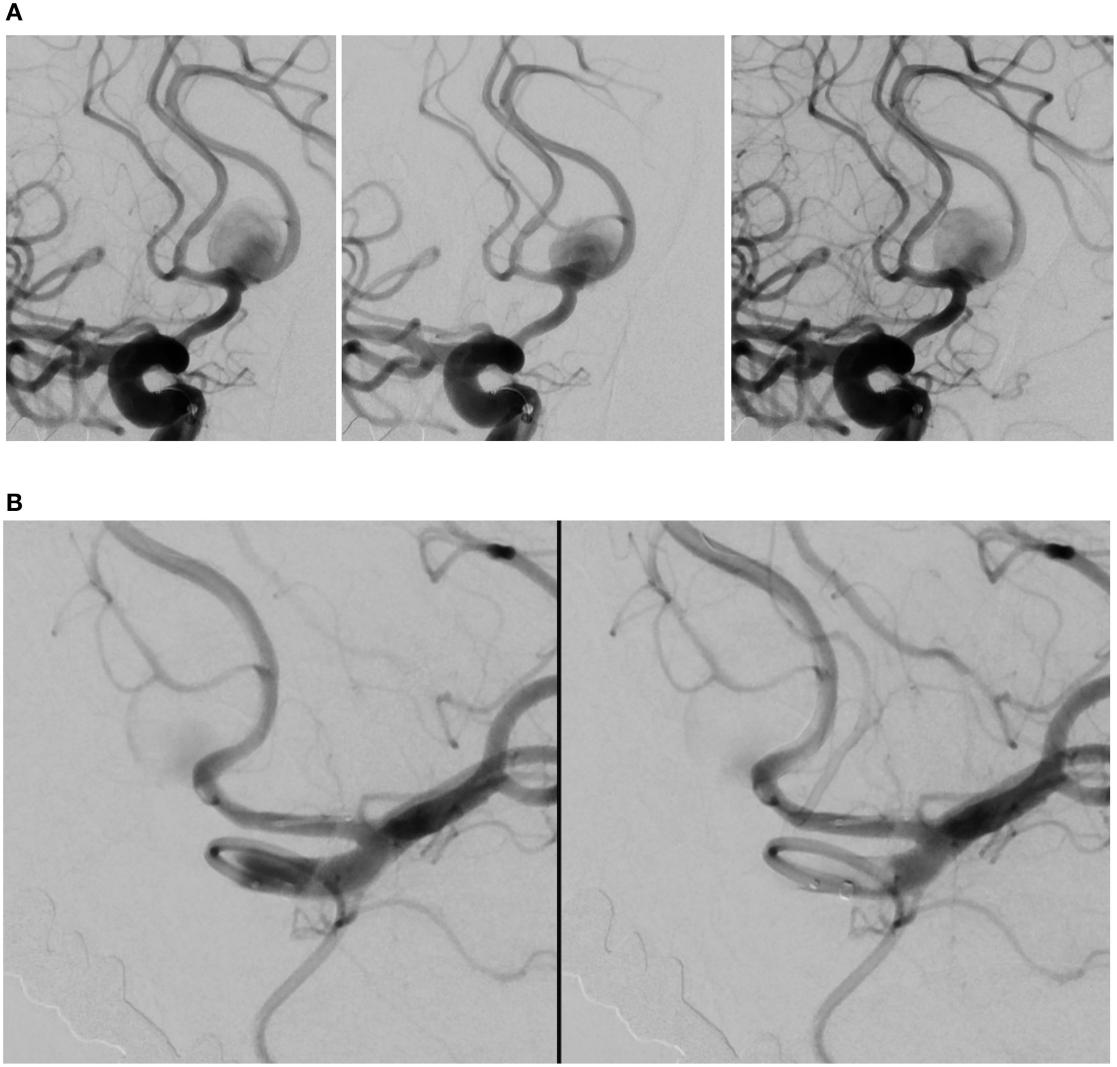
Angiography deployment of SVB stents using kissing technique (stent diameter: 2.75 mm, length: 20 mm). **(A)** Right ICA contrast media (CM) injection following SVB implantation using kissing technique. Opacification of the A2 segment indicates blood flow diversion from the A1 segment into A2 segment, later confirmed by the aneurysm sac filling with CM. **(B)** Left ICA CM in the same patient. The aneurysm sac is less opacified by the CM, where blood flow diversion is immediately apparent.

**Figure 2:**
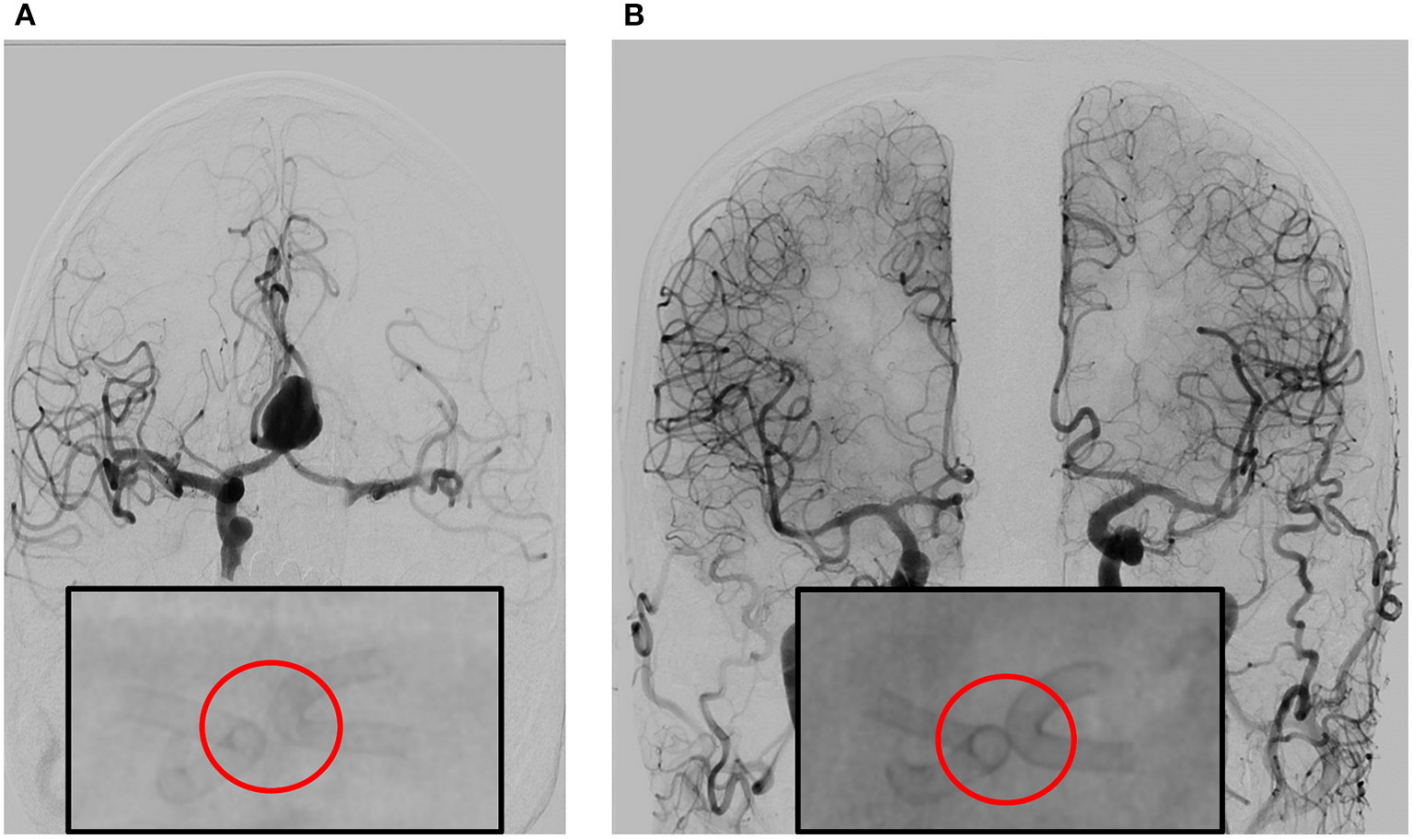
**(A)** Angiography prior to double stent bilateral deployment. Magnified: Angiography shows regular stent profile after the deployment with very small distance between the stents. Deployed on A1-2 angle of the left and right anterior cerebral artery (ACA). **(B)** Follow-up angiography at 6 months, obtained by bilateral contrast media (CM) injection from common carotid artery on the right and left side. The image shows complete exclusion of the aneurysm—which is quite rare—with diversion of the blood flux, in complete separation of intracranial flux of the left and right hemisphere. Magnified: Angiography shows regular stent profile with outer profile of the stent curves getting closer and then coming into contact, resulting in the “kissing” effect. Compared to stent positioning in **(A)**, the image in **(B)** is an indirect sign of shrinking of the aneurysm sac.

For endovascular treatment, a total of 20 FDs were used, all of which were SVB. Adequate aneurysm coverage was achieved with a single FD device in 88.9% (16/18) of patients, including in two patients who had two aneurysms each. Both patients had one aneurysm on the ACoA and the other on the right MCA; in these cases, a single stent was sufficient to cover both aneurysms and the entire dysplastic portion of the parent artery. Two patients (2/18, 11.1%) required multiple FDs; in these cases, each patient had two aneurysms on the ACoA involving the right and left A1-A2 segments. They were each treated with two stents placed with the kissing technique, demonstrated in Figure 1 (13). Follow-up imaging at 6 months for one of these two patients is shown in Figure 2. Five patients with one aneurysm on the ACoA involving the right or left A1-A2 segments were treated with a single stent deployed from the contralateral A2 segment through the ACoA and into the ipsilateral A1, covering the ipsilateral A2 as a side branch. Intervention characteristics are in Table 2.

**Table 2:** Intervention characteristics, outcomes, and follow-up.

	**Frequency**
**Intervention characteristics**	
Complete coverage on first pass	16/18, 88.9%
One FD used	16/18, 88.9%
Two FDs used	2/18, 11.1%
Failure to catheterize	0/18, 0%
Failure of FD deployment	0/18, 0%
Imprecise FD deployment	0/18, 0%
**Complications**	
Mortality	0/18, 0%
Intracerebral bleeding	0/18, 0%
Aneurysm rupture	0/18, 0%
Side branch occlusion	1/18, 5.6%
In-stent thrombosis	1/18, 5.6%
**mRS at discharge**	
0	14/18, 77.8%
1	2/18, 11.1%
2	0/18, 0%
3	2/18, 11.1%
**MRI follow-up at 3 months**	
Complete occlusion	10/15, 66.7%
Small remnant (<10% aneurysmal sac)	3/15, 20.0%
Large remnant (~30% aneurysmal sac)	1/15, 6.7%
Indeterminate	1/15, 6.7%
**Angiography follow-up at 6 months**	
Complete occlusion	10/13, 76.9%
Small remnant (<10% aneurysmal sac)	2/13, 15.4%
Large remnant (~40% aneurysmal sac)	1/13, 7.7%

*Frequencies are reported as (n/N, %). FD, flow diverter; mRS, modified Rankin Scale*.

### Intraprocedural and Clinical Outcomes

Few technical or clinical complications were observed. Failure to catheterize the parent artery did not occur (0/18, 0%)—though in one patient it was necessary to loop the sac with the microguide and catheter—nor did stent deployment failure occur (0/18, 0%). There were no instances of imprecise FD placement with incomplete neck covering, guidewire rupture, dissection of the artery wall, or aneurysm rupture. In all cases (18/18, 100%), slower flow and contrast stagnation were observed in the aneurysmal sac at the end of the procedure, indicating FD placement was technically successful.

No patients experienced intracerebral bleeding or procedure-related death (0/18, 0%). A small minority of patients incurred adverse device-related events, including side branch occlusion (1/18, 5.6%) and in-stent thrombosis (1/18, 5.6%). In one patient with recurrent aneurysm involving the ACoA, acute thrombosis occurred in the middle of the stent following cross-stenting treatment (likely due to the progression of sac thrombosis), but this was fully resolved after administration of a half dose of abciximab calculated based on patient bodyweight. At discharge, 77.8% (14/18) of patients had an mRS of 0, indicating no disability; 11.1% (2/18) had an mRS of 1, indicating light disability; and 11.1% (2/18) had an mRS of 3, indicating moderate disability. Intraprocedural and clinical outcomes are presented in Table 2.

### Follow-Up Imaging

No patients (0/18, 0%) in this study were lost to follow-up; however, three patients (3/18, 16.7%) who were recently treated have not yet had their 3-month follow-up MRI visit, and a total of five patients (5/18, 27.8%) have not yet had their 6-month follow-up angiography visit. All follow-up imaging results are presented in Table 2.

**Figure 3:**
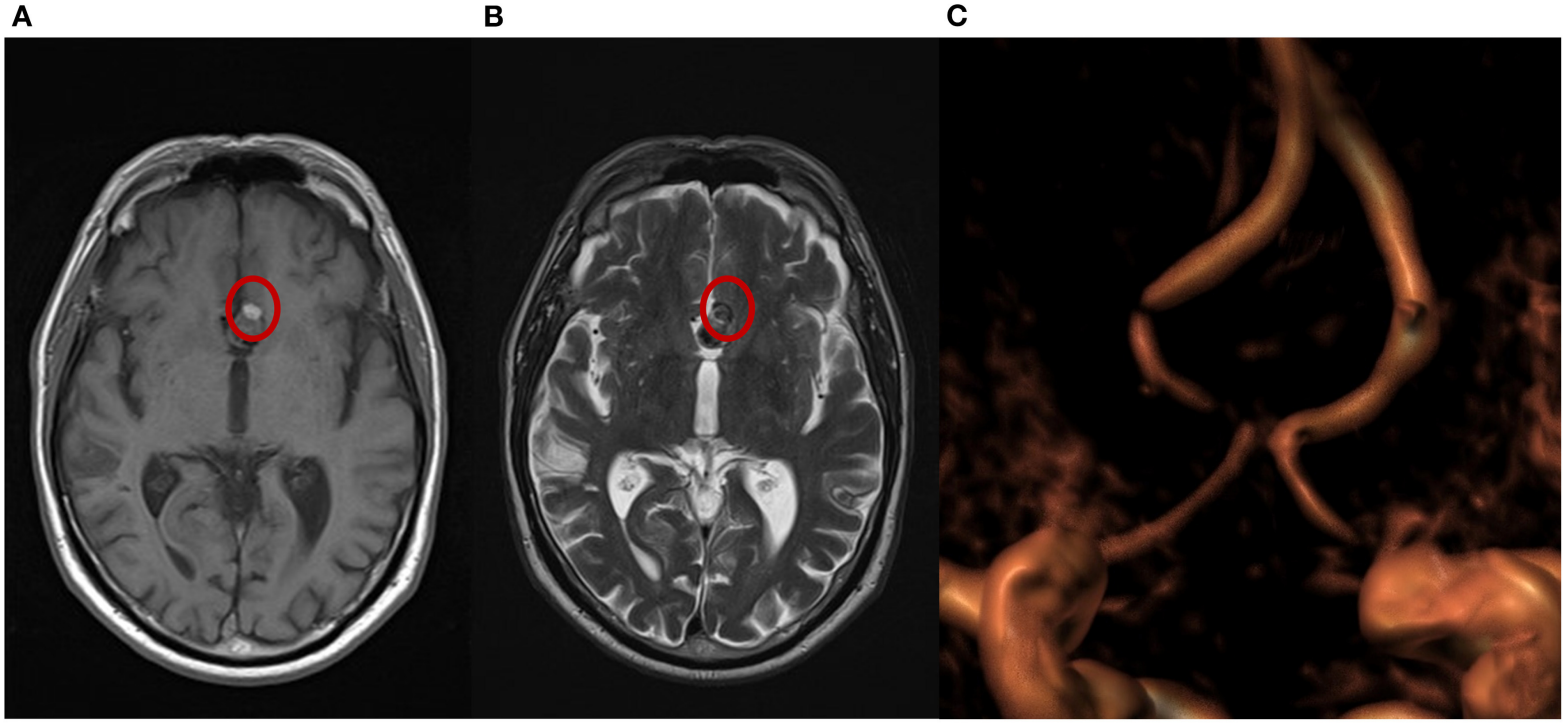
Magnetic resonance imaging (MRI) from a patient with complete occlusion at 3-month follow-up. **(A)** T1 weighted spin echo (T1W SE); a small portion of hyperintensity can be visualized at the bottom of the aneurysm. **(B)** T2 weighted turbo spin echo (T2W TSE); a small portion of isointensity in the same area shows a different grade of cloth maturation in the thrombosis process occurring in the sac after the treatment. **(C)** A 3-dimensional time of flight (TOF 3D) volume rendering image shows no flow in the aneurysm.

Of the 15 patients who have had 3-month MRI follow-up, 66.7% (10/15) had complete occlusion of the aneurysm from the intracranial circulation. In one patient (1/15, 6.7%), MRI examination was indeterminate; angiography was then performed and confirmed complete occlusion of the aneurysm. In 20.0% (3/15) of patients, only a small remnant (<10.0% of original aneurysmal sac) was observed. MRI of the sole patient with a PCA aneurysm—who also had the largest aneurysm in the study (maximum diameter 25 mm)—showed a remnant ~30.0% of the original aneurysm sac, along with increased size of the aneurysm sac, trunk compression, and an associated mRS of 3. Example imaging of a patient with complete occlusion at 3-month follow-up is shown in Figure 3.

As of submission for publication, 13 patients in this study have had 6-month angiographic follow-up.

**Figure 4:**
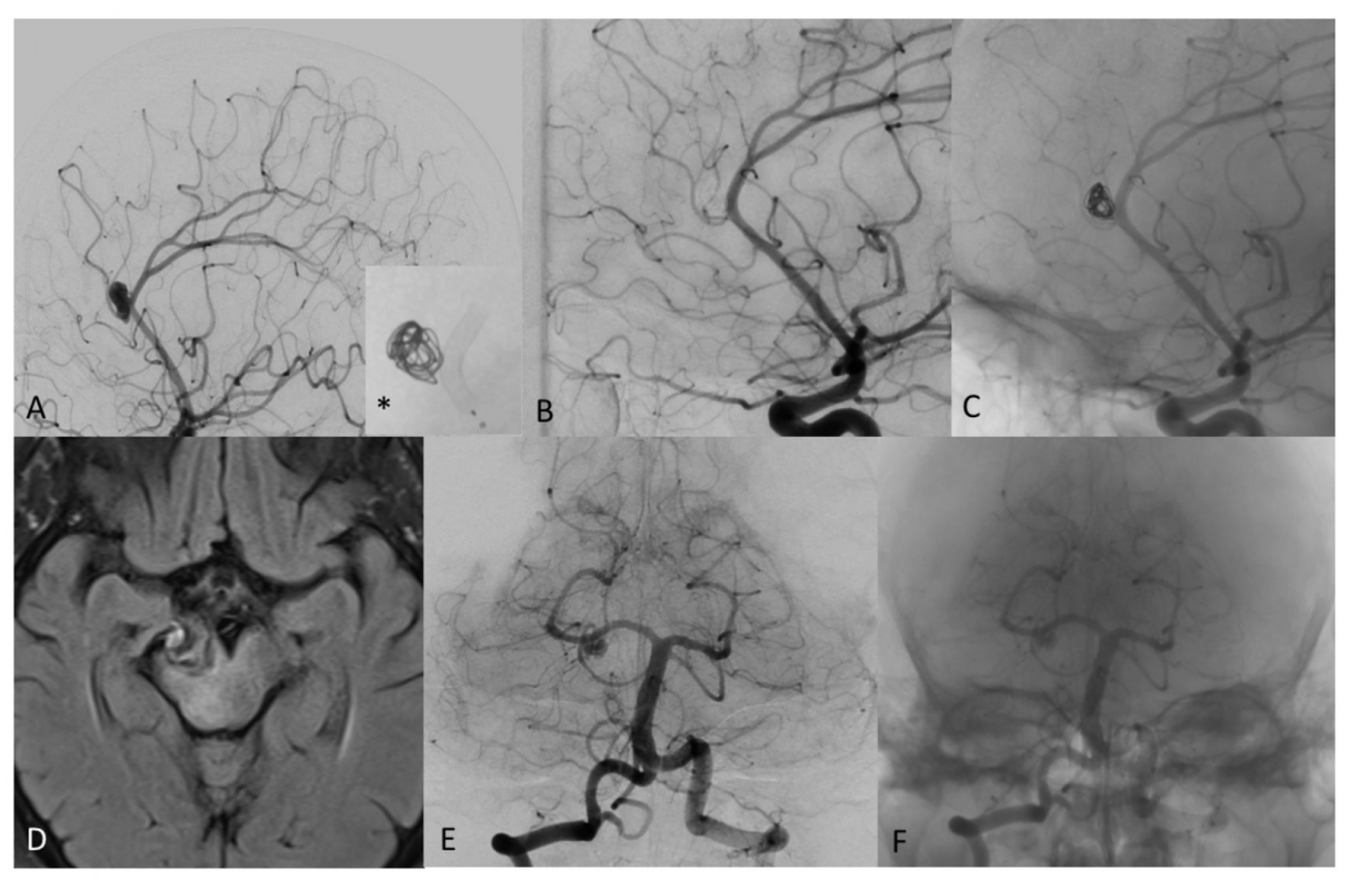
**(A)** Digital subtraction angiography (DSA) of a patient with distal pericallosal-callosomarginal aneurysm treated with non-compact coiling and FD (*), to maintain the patency of callosomarginal artery arising from the neck aneurysm. **(B)** DSA shows complete occlusion of aneurysm sac at 6-month follow-up (stent diameter: 2.25 mm, length: 15 mm). **(C)** Un-subtracted image where it is possible to see the stent and the flow of contrast with the coils of the same patient at 6 months showing complete exclusion of the sac, no contrast filling through the coils and regular patency of treated arteries. **(D)** T2 weighted MRI image of mostly thrombosed giant PCA aneurysm. **(E)** DSA 2-month follow-up after FD treatment with remnant neck patency in patient with significant clinical worsening. **(F)** Un-subtracted image where it is possible to see the stent and the flow of contrast in the proximal part of the neck aneurysm.

**Figure 5:**
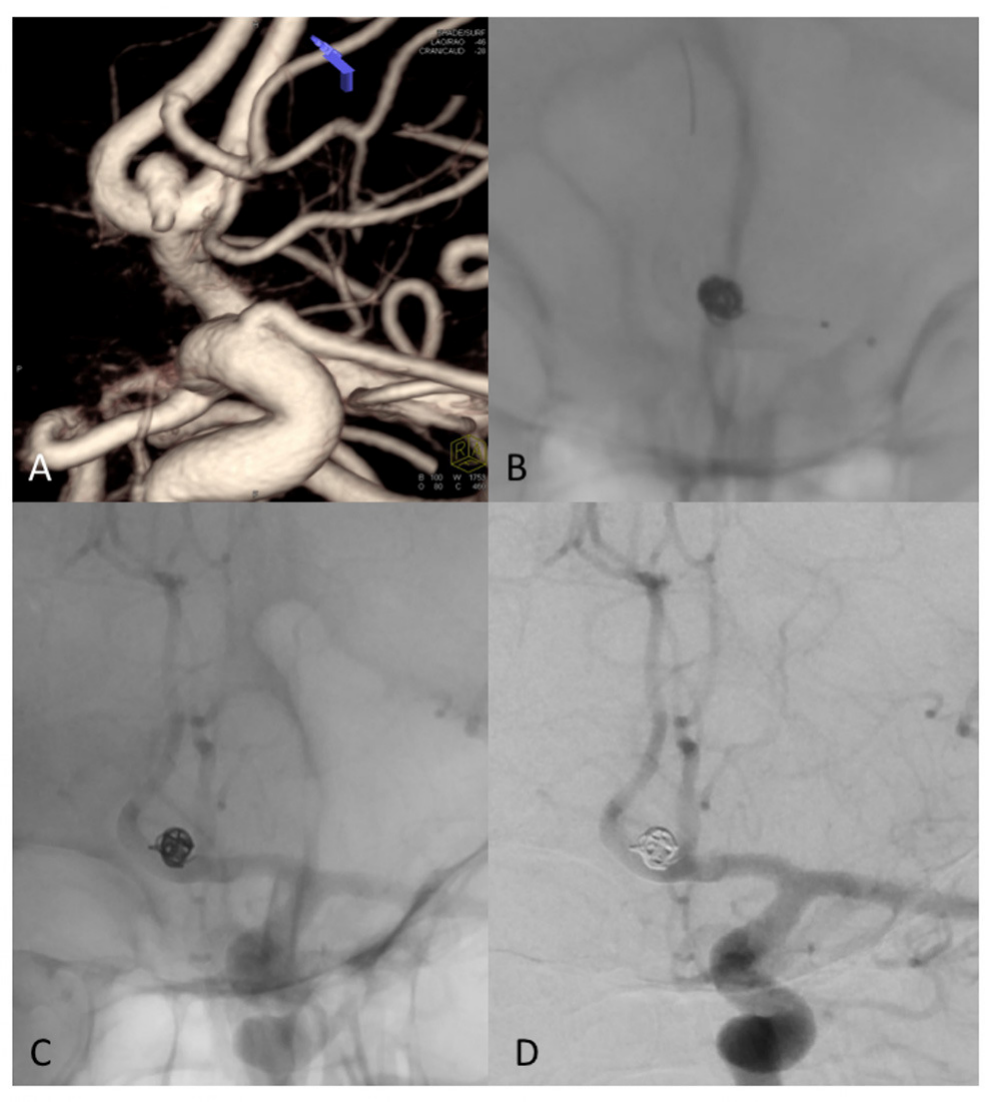
**(A)** 3D Rotational angiography with volume rendering (VR) of ACom complex aneurysm that involving the anterior vessel wall with superior (larger) and inferior (very small, less than an 1 mm) bleb. **(B)** Fluoroscopy shows coils and FD treatment (stent diameter: 2.75 mm, length: 15 mm) of the aneurysm sac. **(C)** Un-subtracted image where it is possible to see the stent and the flow of contrast with the coils of the same patient at 6 months showing complete exclusion of the sac, no contrast filling through the coils and regular patency of treated arteries. **(D)** DSA shows complete occlusion of aneurysm sac at 6-month follow-up.

In 76.9% (10/13) of cases, angiography showed complete occlusion. In 15.4% (2/13) of cases, only a small remnant was observed. Angiography of the patient with the PCA aneurysm showed a remnant at least 40.0% of the original aneurysm sac, with the aneurysm sac further increased in size. No significant stenosis, proximal or distal stent profile deformation, or “fish mouth” effect were observed in any deployed stents in any patient at follow-up angiography. Imaging of a patient with complete occlusion at 6-month follow-up and of PCA aneurysm with a remnant of the original aneurysm sac are shown in Figures 4, 5.

## Discussion

This study showed high rates of technical success and aneurysm occlusion with no mortality and few instances of adverse events related to the SVB. Significantly, many of the aneurysms in this study had unfavorable circumstances based on their specific location or history of recurrence/rupture. Though this case series is limited in size, it demonstrates promising results for the SVB and broadens its evidence base in the literature.

Other than a case report of two patients by Bhogal et al. (9), the present case series represents only the third study to be published regarding the SVB. In Schob's et al. prospective study of 25 patients with 27 aneurysms, they reported no intraprocedural technical complications and a 63.0% complete occlusion rate at 3-month follow-up (14), almost exactly mirroring results from the current study. In Martínez-Galdámez's et al. multicenter retrospective series of 41 patients with 43 aneurysms, they reported somewhat higher rates of intraprocedural complications (5/41, 12.2%), all of which resolved without clinical sequelae, as well as slightly higher rates of post-procedural events (3/41, 7.3%), which included one central venous catheter infection treated with antibiotics, one heavy headache treated with steroids, and one groin pseudoaneurysm managed with compression (10). Long-term follow-up was not available. In this context, results from this case series align with past experiences and show promise for the SVB's continued use.

The SVB is unique in its capability to be delivered via a 0.017-inch catheter; it shares this distinction with only one other FD, the Woven EndoBridge 17 (WEB 17, Sequent Medical, Aliso Viejo, CA, USA) (4). In two recent retrospective studies, the WEB 17 showed results for safety and feasibility that are comparable to those of the current study. In Maurer's et al. (15) study of 124 treated aneurysms, they reported 0.0% (0/124) mortality, 4.8% (6/124) thromboembolic or hemorrhagic complications resulting in worse clinical outcome for 1.6% (2/124) of patients, 76.1% (70/92) complete occlusion at 3 months, and 78.0% (32/41) complete occlusion at 12 months. Likewise, Goertz et al. (16) found 0.0% (0/28) mortality, 5.3% (2/38) thromboembolic complications causing neurologic complications in 2.6% (1/38) of patients, and 57.9% (22/38) complete occlusion immediately following treatment with the WEB 17. Although time and additional studies are needed to expand the evidence base for these newer FDs, preliminary results indicate the safety and feasibility of both the SVB and WEB 17 for aneurysm treatment.

Delivery of an FD device through the smallest available catheters is crucial to treat aneurysms arising from distal, small-caliber vessels. While it is unclear whether flow diversion treatment in small vessels is more likely to result in clinical complications (17), evidence indicates that interventionalists are more likely to be met with intraprocedural technical difficulties in these cases, such as navigating increased vessel tortuosity (18, 19). It is notable that in this study, all aneurysms were located on small intracranial arteries, many of which had less than ideal circumstances; a majority of aneurysms were on the ACoA, involving the right or left A1-A2 segments, and a few aneurysms recurred after coil embolization. The deliverability of the SVB via a 0.017-inch microcatheter provided interventionalists enhanced navigability and allowed comparatively simple access to challenging segments of the intracranial arteries. In this way, SVB implantation was performed without technical difficulty in any cases, even in rather peripheral locations.

Additionally, when using FDs in small vessels, it is imperative to understand and consider the effects of oversizing on the FD. Oversizing may result in lengthening of the device, subsequently increasing its porosity and likelihood of aneurysm persistence (6, 7). It should be highlighted that in this study, measurements were not made on volume rendering images; rather, they were made on the axial plane after reconstruction of the image using the “Vessel analysis” software. By using this technique, interventionalists were able to make precise measurements of the involved vessels and ultimately avoid injury due to stent oversizing.

## Study Limitations

Limitations of this study include its retrospective, non-randomized design, limited sample size as a case series, and short follow-up period. Progressive aneurysm occlusion over time is common after flow diversion, so it is expected for occlusion rates to increase as more extensive follow-up is available. Larger, prospective, randomized studies with longer-term follow-ups are needed to corroborate the effectiveness of this treatment method and its comparison to other devices.

## Conclusion

This study demonstrated that the use of SVB for treatment of intracranial aneurysms is feasible and safe, with no procedural difficulties, no mortality, and few device-related adverse events. Future studies with larger samples and randomized treatment allocation will shed light on the comparative efficacy of SVB and other FDs.

## Data Availability Statement

The raw data supporting the conclusions of this article will be made available by the authors, without undue reservation.

## Ethics Statement

Ethical review and approval was not required for the study on human participants in accordance with the local legislation and institutional requirements. The patients/participants provided their written informed consent to participate in this study.

## Author Contributions

VG and AD validated the study, conducted the formal analysis and investigation, and participated in writing the original draft of the manuscript. VG conceptualized the study, designed the methodology, provided the necessary software, provided study resources, curated the data, reviewed and edited the manuscript, and provided study supervision and project administration. All authors contributed to study visualizations.

## Conflict of Interest

The authors declare that the research was conducted in the absence of any commercial or financial relationships that could be construed as a potential conflict of interest.

## Abbreviations

ACA: Anterior cerebral artery
ACoA: Anterior communicating artery
FD: Flow diverter
FDA: Food and Drug Administration
MCA: Middle cerebral artery
MRI: Magnetic resonance imaging
mRS: Modified Rankin Scale
PCA: Posterior cerebral artery
SAH: Subarachnoid hemorrhage
SVB: Silk Vista Baby
WEB 17: Woven EndoBridge 17.
